# RGDS-Modified Superporous Poly(2-Hydroxyethyl Methacrylate)-Based Scaffolds as 3D In Vitro Leukemia Model

**DOI:** 10.3390/ijms22052376

**Published:** 2021-02-27

**Authors:** Hana Svozilová, Zdeněk Plichta, Vladimír Proks, Radana Studená, Jiří Baloun, Michael Doubek, Šárka Pospíšilová, Daniel Horák

**Affiliations:** 1 Center of Molecular Medicine, Central European Institute of Technology, Masaryk University, Kamenice 5, 625 00 Brno, Czech Republic; svozilova.hana@gmail.com (H.S.); radana.studena@gmail.com (R.S.); jiri.baloun@ceitec.muni.cz (J.B.); doubek.michael@fnbrno.cz (M.D.); sarka.pospisilova@ceitec.muni.cz (Š.P.); 2 Department of Internal Medicine—Hematology and Oncology, University Hospital Brno and Faculty of Medicine, Masaryk University, Jihlavská 20, 625 00 Brno, Czech Republic; 3 Institute of Macromolecular Chemistry, Czech Academy of Sciences, Heyrovského nám. 2, 162 06 Prague, Czech Republic; plichta@imc.cas.cz (Z.P.); proks@imc.cas.cz (V.P.)

**Keywords:** poly(2-hydroxyethyl methacrylate), 3D scaffold, RGDS, chronic lymphocytic leukemia, B cell survival

## Abstract

Superporous poly(2-hydroxyethyl methacrylate-*co*-2-aminoethyl methacrylate) (P(HEMA-AEMA)) hydrogel scaffolds are designed for in vitro 3D culturing of leukemic B cells. Hydrogel porosity, which influences cell functions and growth, is introduced by adding ammonium oxalate needle-like crystals in the polymerization mixture. To improve cell vitality, cell-adhesive Arg-Gly-Asp-Ser (RGDS) peptide is immobilized on the *N*-(γ-maleimidobutyryloxy)succinimide-activated P(HEMA-AEMA) hydrogels via reaction of SH with maleimide groups. This modification is especially suitable for the survival of primary chronic lymphocytic leukemia cells (B-CLLs) in 3D cell culture. No other tested stimuli (interleukin-4, CD40 ligand, or shaking) can further improve B-CLL survival or metabolic activity. Both unmodified and RGDS-modified P(HEMA-AEMA) scaffolds serve as a long-term (70 days) 3D culture platforms for HS-5 and M2-10B4 bone marrow stromal cell lines and MEC-1 and HG-3 B-CLL cell lines, although the adherent cells retain their physiological morphologies, preferably on RGDS-modified hydrogels. Moreover, the porosity of hydrogels allows direct cell lysis, followed by efficient DNA isolation from the 3D-cultured cells. P(HEMA-AEMA)-RGDS thus serves as a suitable 3D in vitro leukemia model that enables molecular and metabolic assays and allows imaging of cell morphology, interactions, and migration by confocal microscopy. Such applications can prospectively assist in testing of drugs to treat this frequently recurring or refractory cancer.

## 1. Introduction

Chronic lymphocytic leukemia (CLL) is the most common adult blood cancer in Caucasians. It is characterized by malignancy of morphologically mature CD5 positive B cells (B-CLLs), which results in the proliferation and accumulation of the neoplastic B cells in the blood, bone marrow, and secondary lymphoid tissues [1]. The nature of CLL is highly heterogeneous in terms of clinical course and overall survival. Some patients remain asymptomatic and survive for decades, whereas others can experience an aggressive course of the disease surviving for 3 to 5 years. Promisingly, novel drugs and their combinations have been introduced for CLL treatment [2]. While these treatments undeniably improve patient prognosis and can lead to long-lasting remissions, relapses still occur. Despite the worldwide effort to develop a cure for CLL, the disease is mostly incurable and the mechanisms describing CLL biology, relapses, and refractoriness have not been fully resolved [3,4]. Consequently, CLL research has been mostly focused on elucidating the molecular mechanism of the disease, while the search for the right treatment persists.

In current studies of cancer pathophysiology and drug development, the focus has been laid on 3D in vitro models [5,6,7]. 3D culture systems, rather than conventional ones (i.e., monolayers or suspension cultures), allow cells to grow, migrate, interact, and respond to various stimuli analogously to real in vivo behavior [8]. In vitro studies of CLL are also complicated by the fact that in the conventional cell culture, primary B-CLLs, which are dependent on their natural microenvironment, quickly undergo apoptosis and are not able to establish a stable cell line [9]. Consequently, novel bone marrow-like scaffolds that mimic the CLL microenvironment are being developed to reveal the mechanisms of CLL biology [10,11]. In order to fully understand the 3D in vitro cell interactions under different conditions, the development of novel 3D CLL models is thus of utmost importance. 

Nowadays, 3D culture systems offer a vast number of options, including various scaffold-free or scaffold-based approaches [12,13]. A plethora of hydrogels or other materials based on both natural and synthetic polymers have been described for the scaffold design, including alginate, chitosan, collagen, hyaluronic acid, poly(lactic or glycolic acid), poly(hydroxybutyrate-*co*-hydroxyvalerate), poly(ester urea), poly(ester amide), poly(ethylene glycol), polyanhydrides, etc. [14,15,16]. Hydrogel scaffolds have an advantage in that they mimic an extracellular matrix, enabling the survival, proliferation, differentiation, and migration of the cells. This is due to biocompatibility (nontoxicity) of the hydrogels, the softness of which resembles living tissue, interconnecting pores, high water retention, efficient transport of oxygen and nutrients, etc. [17,18]. Among the hydrogels, poly(2-hydroxyethyl methacrylate) (PHEMA) plays a special role due to its tissue-like mechanical compliance, elasticity, mass transfer properties, and long history of successful applications in medicine and tissue engineering as soft contact lenses, surgical implants, and drug delivery vehicles [19,20]. Another advantage of PHEMA is that it can be easily fabricated into various configurations and shapes and conveniently chemically modified, e.g., by copolymerization of 2-hydroxyethyl methacrylate (HEMA), with various reactive comonomers. Moreover, PHEMA is partly transparent in water to light, enabling inspection of cell models with optical microscopic techniques. 

When choosing artificial 3D matrices for B-CLLs, it is crucial to fulfill many requirements; most importantly, the scaffolds should be geometrically similar to the B-CLL microenvironment. In addition, the viability of B-CLLs strictly depends on the presence of essential interactions [9]. Thus, natural conditions have to be simulated by the addition of growth factors and/or by culture with cells which typically occur in the pores of the bone marrow, e.g., bone marrow stromal cells (BMSCs), T cells, or nurse-like cells [21]. While physical cell–cell contacts are necessary for BMSCs and B-CLLs [22], the presence of stimulating T cells can be advantageously replaced by the growth medium with CD40 ligand (CD40L) and interleukin 4 (IL-4), etc. Not only do the aforementioned interactions contribute to evading the B-CLLs from both in vivo and in vitro apoptoses, but they also stimulate the B-CLLs to boost their proliferation [23]. 

The aim of this study was to develop a 3D hydrogel scaffold that is biocompatible, partly transparent, and contains sufficiently large pores to accommodate the cells. The hydrogel was based on superporous poly(2-hydroxyethyl methacrylate-*co*-2-aminoethyl methacrylate) (P(HEMA-AEMA)), which was modified with the cell adhesion Ac-CGGGRGDSGGGY-NH_2_ (RGDS) peptide. The superporosity was reached by needle-shaped ammonium oxalate as a porogen and a final polymer was denoted as P(HEMA-AEMA)-RGDS. Another objective of the study was to optimize a suitable 3D system that resembles the natural environment of the primary B-CLLs, i.e., the trabecular bone, where hematopoiesis occurs and the leukemic B cells are present. Based on the results achieved with other 3D in vitro cancer models [24,25], our system should enable us to predict the sensitivity of B-CLLs to various treatments more accurately than the 2D cell culture. The final goal was to mimic cell–cell interactions by introducing the interaction partners, i.e., BMSCs or soluble factors produced by T cells, into the in vitro microenvironment. 

## 2. Results

### 2.1. Poly(2-Hydroxyethyl Methacrylate-co-2-Aminoethyl Methacrylate) (P(HEMA-AEMA)) Scaffolds

**Figure 1:**
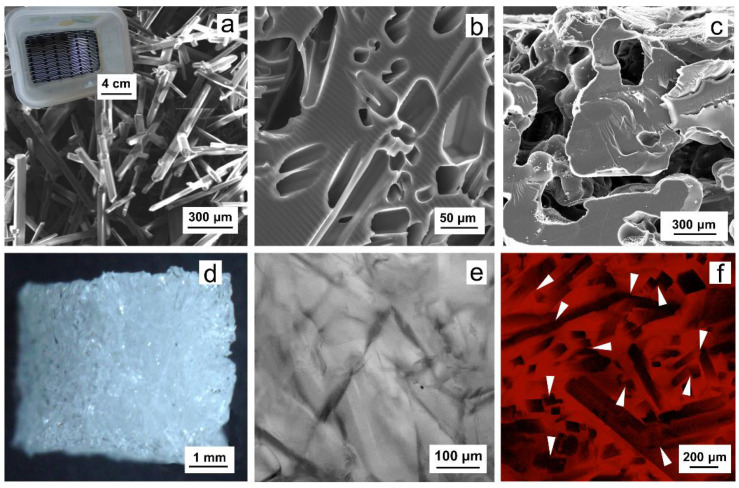
Scanning electron microscopy (SEM) micrographs of (**a**) ammonium oxalate crystals (inset: sieve for screening the needles), (**b**,**c**) superporous poly(2-hydroxyethyl methacrylate-*co*-2-aminoethyl methacrylate) (P(HEMA-AEMA)) hydrogel, and (**d**) light and (**e**,**f**) confocal micrograph of a hydrogel cube. (**f**) Single plane of hydrogel stained with AlamarBlue™, white arrows point to pore intersections. HEMA—2-hydroxyethyl methacrylate; AEMA—2-aminoethyl methacrylate.

P(HEMA-AEMA) scaffolds were obtained by 2,2′-azobis(2-methylpropionitrile) (AIBN)-initiated radical polymerization of 2-hydroxyethyl methacrylate (HEMA), ethylene dimethacrylate (EDMA), and 2-aminoethyl methacrylate (AEMA) in the presence of ammonium oxalate crystals as a porogen. The crystals were 30–60 μm thick, 0.3–2 mm long, and polydisperse in size (Figure 1a). In the synthesis, content of the ammonium oxalate crystals in the polymerization mixture during sedimentation amounted to 43 vol.%. After completion of the polymerization, the ammonium oxalate was removed from the polymer by washing with water, leaving interconnected pores in the hydrogel as reprints of the original crystals (Figure 1b–f); as a result, the pores were close to the structure of trabecular bone [26,27]. The interconnectivity of pores was documented in Figure 1c,f. P(HEMA-AEMA) hydrogels were fabricated in the form of cylinders by polymerization of the monomer mixture in injection syringes. The cylinders were then portioned to small cubes to facilitate future cell penetration (Figure 1d). The resulting P(HEMA-AEMA) contained 0.35 wt.% of N according to the elemental analysis, corresponding to 0.25 mmol of amino groups per g. This is more than the number of the groups in AEMA added in the polymerization feed. The discrepancy can be explained by the presence of residual nitrile groups originating from AIBN initiator. According to scanning electron microscopy (SEM) micrographs of the superporous P(HEMA-AEMA) hydrogels in the dry state, the diameters of the pores reached 15–60 μm (Figure 1b), which approximately corresponded to the size of original crystals. P(HEMA-AEMA) hydrogels swelled in water, where their pore sizes slightly increased (Figure 1f). It is worth noting that our earlier PHEMA-based scaffolds prepared with ammonium oxalate as a porogen reached water regain of 1.83 mL/g [28].

### 2.2. Immobilization of RGDS Peptide on P(HEMA-AEMA)-RGDS Scaffolds

**Figure 2:**
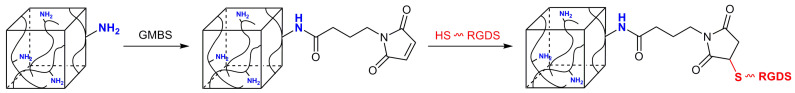
Scheme of immobilization of Ac-CGGGRGDSGGGY-NH_2_ (RGDS) peptide on P(HEMA-AEMA) hydrogel via *N*-(γ-maleimidobutyryloxy)succinimide (GMBS) activation.

Since cell adhesion and motility strongly depend on the interactions between cells and scaffold, it is preferable to modify its surface with an adhesive peptide, although the cells alone can colonize the neat (unmodified) PHEMA scaffold used as cell support. P(HEMA-AEMA) properties were strengthened by *N*-(γ-maleimidobutyryloxy)succinimide (GMBS) activation and RGDS peptide to enhance cell adhesion and spreading on the scaffold. While the *N*-hydroxysuccinimide ester of GMBS was covalently conjugated with amino groups of the P(HEMA-AEMA) scaffold at pH 7.4 to form amide bonds, the maleimide reacted with thiol groups of RGDS at pH 6.8 to produce stable thioether bonds (Figure 2). The amount of immobilized RGDS was determined in our previous report, where 65 ± 10 µg of RGDS was found per P(HEMA-AEMA)-RGDS disc (i.e., 2.16 ± 0.9 × 10^−13^ mol of RGDS per cm^2^) according to quantitative radioassay analysis using ^125^I-labeled peptide [29]. 

### 2.3. 3D Culture of Leukemia B Cells on P(HEMA-AEMA)-RGDS Scaffolds

The optimal cell seeding method, cell adhesion, viability, and metabolic activity on the P(HEMA-AEMA)-RGDS hydrogels were determined from confocal microscopy micrographs and AlamarBlue™ assay. Coculture experiments were only studied by confocal microscopy, as the AlamarBlue™ assay does not distinguish the contribution of each cell type to the overall metabolic activity. As the hydrogels were partly transparent in medium, they allowed the imaging of even the cells seeded deep inside of the matrix (≤300 μm). 

Each method of investigation and its conditions were first optimized in terms of the corresponding cell lines, which are immortal and resilient, compared to primary B-CLLs. Since BMSCs cultured with primary B cells mimic the natural microenvironment of the bone marrow, it was important to ensure that both the BMSCs and the B-CLLs penetrate the P(HEMA-AEMA)-RGDS scaffold and proliferate there. Consequently, the emphasis was laid on the cell culture conditions of both cell types. Coculture of BMSCs was further optimized preferably with murine BMSC lines since they resemble primary BMSCs in terms of phenotypic characters [30] and trigger more homogenous responses compared to primary or immortalized human BMSCs [31].

**Figure 3:**
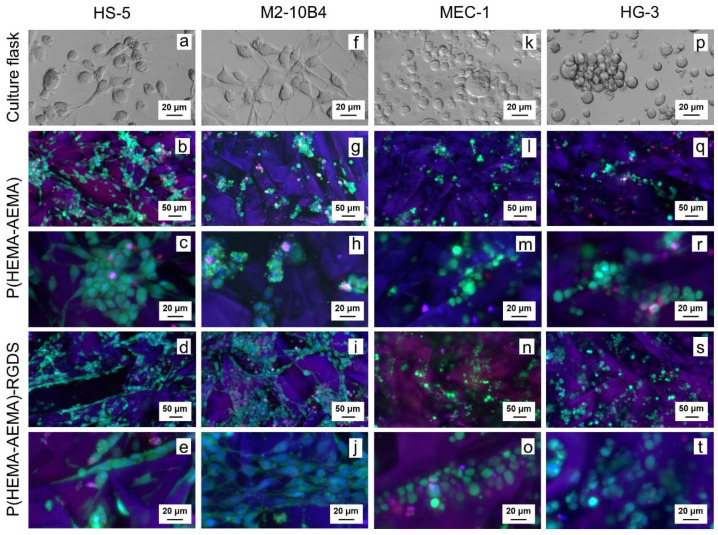
Confocal micrographs of P(HEMA-AEMA)-RGDS scaffolds seeded with (**a**–**e**) HS-5, (**f**–**j**) M2-10B4, (**k**–**o**) MEC-1, (**p**–**t**) HG-3 cell culture for 24 h. (**a**,**f**,**k**,**p**) Transmitted light; (**b**–**e**, **g**–**j**, **l**–**o**, **q**–**t**) live and dead cells and nuclei stained by calcein acetoxymethyl ester (AM) (green), propidium iodide (red), and Hoechst 33342 (blue), respectively.

Cell morphology, distribution, and viability of the P(HEMA-AEMA)-RGDS scaffolds were determined by confocal microscopy (Figure 3). The HS-5 cells were mostly of polygonal shapes with fusiform morphologies in all culture types (Figure 3a–e). M2-10B4 also adhered to surface in flasks (Figure 3f) and RGDS-modified scaffolds (Figure 3i,j), but not in hydrogels without surface modifications (Figure 3g,h). The morphologies of both tested B-CLL lines MEC-1 (Figure 3k–o) and HG-3 (Figure 3p–t) corresponded with the supplier’s description: round cells growing in suspension, MEC-1 being slightly adherent [32] and HG-3 partially forming clumps [33]. All studied cell lines manifested homogenous distribution within scaffold pores (Figure 3b,d,g,i,l,n,q,s).

**Figure 4:**
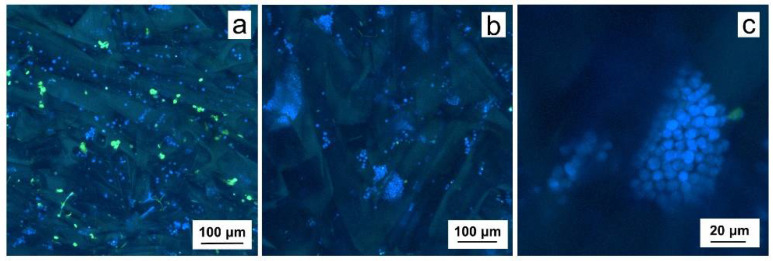
Confocal micrographs of (**a**) P(HEMA-AEMA) and (**b**,**c**) P(HEMA-AEMA)-RGDS scaffolds seeded with primary CD5 positive B cells (B-CLLs) for 24 h. B-CLLs and dead cells were stained by CellTrace™ violet (blue) and Sytox (green), respectively.

Compared to primary B-CLL cells cultured on P(HEMA-AEMA) (Figure 4a), more cells, larger clumps and higher cell viability was observed on the P(HEMA-AEMA)-RGDS scaffolds (Figure 4b,c). While only 75% viability was calculated for P(HEMA-AEMA)-cultured cells, 95% (Figure 4b) or 98% (Figure 4c) live B-CLLs were seen in RGDS-modified hydrogels.

Optimal cell seeding concentration: The image analysis, i.e., cell counts and viability, assisted in the optimization of cell seeding concentration, which differed depending on the cell type. Multiple repetitions of the experiments showed highly heterogeneous results; therefore, the resulting optimal concentration is given in an interval in which the cell counts and viability were the highest. To enhance the probability of cell accommodation in the hydrogels, they were incubated with an excess of the cells. The adherent cell lines HS-5 and M2-10B4 generally required lower concentrations (3–4 million/mL) compared to the HG-3 and MEC-1 cell lines growing in the suspension (15–25 million/mL). Primary B-CLL cells were seeded in the highest concentration (~50 million/mL) to compensate for low proliferation and high rate of apoptosis, which is not seen in the immortalized cell lines. When coculture was performed, cell densities of 3 million/mL BMSCs and 50 million/mL B-CLLs were used.

**Figure 5:**
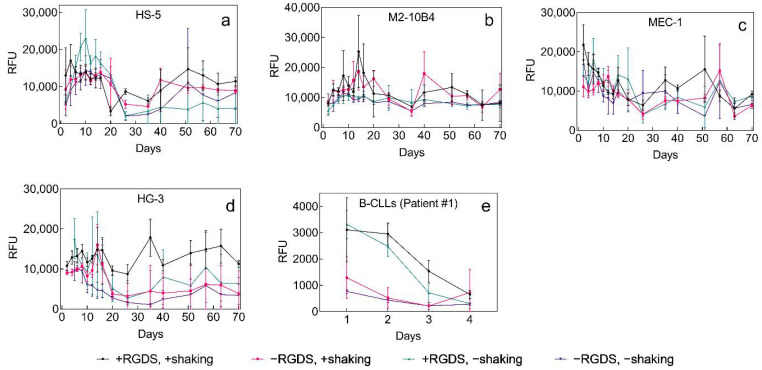
Time dependence of (**a**) HS-5, (**b**) M2-10B4, (**c**) MEC-1, (**d**) HG-3, and (**e**) primary B-CLL metabolic activity (patient No. 1, Table S1 in Supporting Materials (SMs)) in P(HEMA-AEMA) and P(HEMA-AEMA)-RGDS scaffolds without or with shaking to promote the medium flow. Metabolic activity was quantified by AlamarBlue™ assay for 70 days. RFU—relative fluorescence units; n = 3. Vertical bars in the graphs denote 95% confidence interval. Statistical analysis (Table S2) by Statistica revealed that metabolic activity was significantly affected by shaking in (**a**) HS-5 (*p* < 0.001) and (**b**) M2-10B4 (*p* < 0.01) cell lines and by RGDS surface modification in (**e**) primary B-CLLs (*p* < 0.001). No other significant differences were observed.

Long-term cell line culture. Both adherent (Figure 5a,b) and suspension cells (Figure 5c–e) were cultured in the P(HEMA-AEMA) hydrogel to prove its capability of long-term cell accommodation, which is required for coculture of the primary B-CLLs and BMSCs. AlamarBlue™ assay was used to determine the metabolic activity of cells on the P(HEMA-AEMA) as well as P(HEMA-AEMA)-RGDS scaffolds during 70 days in media in both static and dynamic settings.

All studied cell lines demonstrated the ability to metabolize under the selected conditions for the whole period of 70 days. Even though their metabolic activities occasionally dropped, the cell lines always recovered their rates to a various nonzero extent. Irreversible drop in metabolic activity was only observed in primary B-CLLs, whose metabolic rates decreased after 4 days of seeding (Figure 5e). Even though B-CLLs were seeded at the same density, their initially observed metabolic activity differed. The most homogenous metabolic activity with the minimum number of fluctuations was seen in M2-10B4 cells, which makes them favorable for coculture with primary B-CLLs (Figure 5b).

Effect of medium flow and modification of P(HEMA-AEMA) with RGDS: A significant effect of medium flow was only seen in adhesion cell lines, where the motion of plates positively influenced cellular metabolic activity. In primary or immortalized B-CLL cells, the shaking did not stimulate metabolic activity of cells (Table S2). Interestingly, statistical analysis revealed a significant difference (*p* < 0.001) between the metabolic rate of B-CLLs cultured on the unmodified and RGDS-modified scaffolds; the latter hydrogel supported much higher metabolic rates. These results corresponded with higher adhesion and survival-supporting capacities of the RGDS-modified scaffolds observed by confocal microscopy (Figure 5d,e).

**Figure 6:**
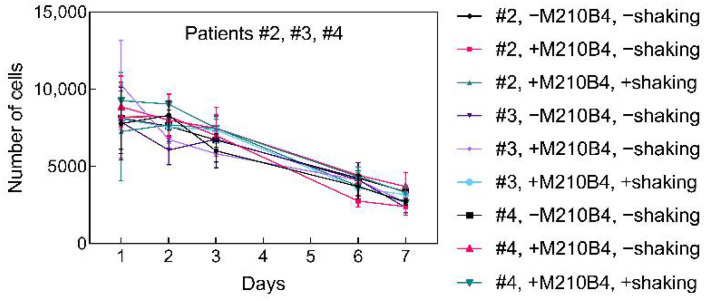
Time dependence of a number of viable B-CLLs of 3 different patients (No. 2–4; Table S1) seeded in the P(HEMA-AEMA)-RGDS hydrogel for 7 days and cultured in the presence or absence of bone marrow stromal cells (BMSCs) with or without supporting medium flow; n = 3. Vertical bars in the graphs denote 95% confidence interval. Statistical analysis (Table S3) by computing environment R; no significant differences were observed.

**Figure 7:**
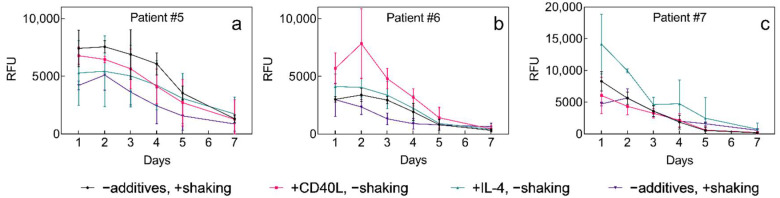
Time dependence of B-CLL metabolic activity of (**a**–**c**) three different patients (No. 5–7, respectively; Table S1) cultured with M2-10B4 cells in the P(HEMA-AEMA)-RGDS hydrogels with or without the addition of soluble IL-4 (10 ng/μL) and CD40L (1 μg/μL). RFU—relative fluorescence units; n = 3. Vertical bars in the graphs denote 95% confidence interval. Statistical analysis (Table S4) by computing environment R; no significant differences were observed.

Prolonged survival of 3D-cultured primary B-CLLs was aimed for by introducing interaction partners into the microenvironment, i.e., either M2-10B4 cells (Figure 6), or soluble IL-4 (5 ng/μL), or CD40L (1 μg/μL) (Figure 7). The delivery of the nutrients was supported by shaking the plates and its effect on cell survival was studied as well. As cells of each individual might respond differently to specific external stimuli, cells from multiple patients were studied [34]. Patients selected for B-CLL culture had different levels of leukocytosis and carried the genetic burden of various severities resulting in adverse clinical implications (Table S1).

The overall B-CLL survival did not differ among selected patients and did not correlate with selected patients’ characteristics. Coculture with M2-10B4 in P(HEMA-AEMA)-RGDS scaffolds had no statistically significant impact on B-CLL survival, which was not improved even by shaking the plates (Figure 6; Table S3). The same applied for soluble additives IL-4, as well as for CD40L (Figure 7a–c; Table S4). On the other hand, shaking of the scaffolds reduced metabolic cell activity in all studied patients with probability *p* = 0.052.

**Figure 8:**
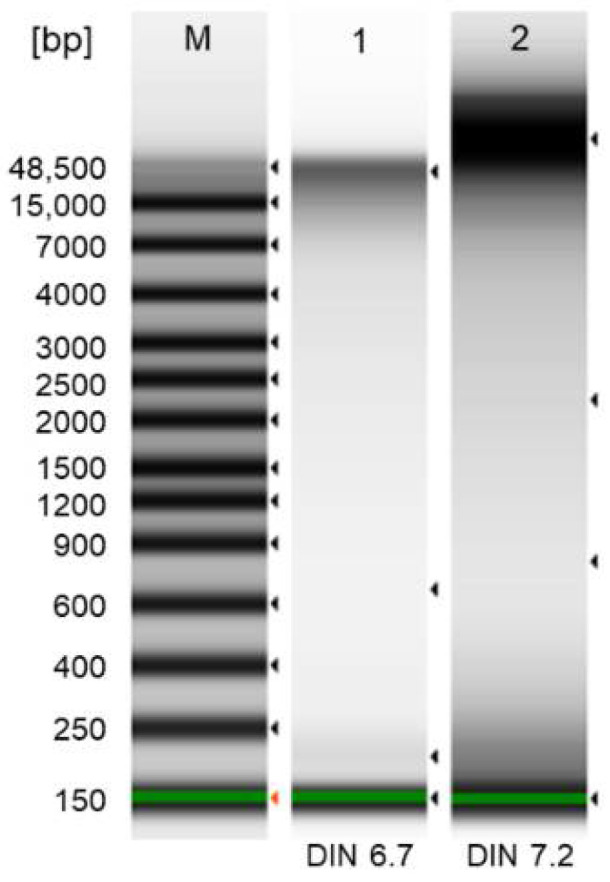
Electropherograms of ladder (1; standard), DNA isolated from B-CLLs cultured in P(HEMA-AEMA)-RGDS hydrogel (2), and DNA isolated from whole blood by MagCore genomic DNA whole blood kit (3; RBC Bioscience; Taipei, Taiwan; positive control). Electropherograms were obtained using a TapeStation 4200 system. DIN—DNA integrity number.

Isolation of DNA: A protocol for isolation of DNA from 3D-cultured B-CLLs was optimized to introduce a methodology for prospective analysis of genetic mutations in time. Let us note that it is crucial to distinguish genetic information of the BMSCs and B-CLLs as both were cocultured. The coculture system of two cell types is inevitable since the protection by BMSCs can only be achieved via cell–cell contacts, not when the B-CLLs are separated from the BMSCs, e.g., by micropore filters [22,31]. Because of this, the cells were directly lysed on the 3D matrices, with the BMSCs and B-CLLs being of murine and human origin, respectively. DNA was successfully isolated within 24 h after B-CLL seeding on the superporous P(HEMA-AEMA)-RGDS scaffold using a FastDNA sample spin kit for soil. The amount of DNA isolated from three hydrogels reached 1.68 μg, with the DNA integrity being 6.7, which is comparable with the integrity achieved after DNA isolation from the whole blood by the automated protocol (Figure 8).

## 3. Discussion

PHEMA was selected for the preparation of superporous hydrogel scaffolds for long-term culture of leukemic cells. To increase the cell adhesive properties, the surface of PHEMA was modified with RGDS peptide via the GMBS coupling reaction [29]. In this report, PHEMA was crosslinked with a small amount of crosslinking agent (EDMA; 1 wt.%) to improve the mechanical properties and hinder the formation of microgels, which would compromise the cell experiment evaluation. In addition to EDMA, the limited amount of AEMA (1 wt.%) was used as a comonomer to introduce amino groups necessary for future reaction with GMBS and subsequent immobilization of RGDS peptide via SH groups (Figure 2). Because effective cell seeding into the hydrogel matrices requires the large pore size, inorganic crystals served as a porogen added in the polymerization feed; they are easily available and their size was regulated by controlling the crystallization conditions. Among various crystal shapes and sizes, in particular, ammonium oxalate needles (Figure 1a) have the advantage of producing continuous pores, in contrast to, e.g., sodium chloride, which is in the form of cubes. To achieve a ~1–5 mm length and prevent breaking of crystalline needles, a special sieve with longitudinal holes made on a 3D printer was used to classify the crystals (inset in Figure 1a). Ammonium oxalate has the additional advantage of introducing connectivity of the pores in places of contacts between the crystals, especially when the crystals are oriented in various directions (Figure 1c,f). These pore interconnections are important for prospective proper nutrient transport, cell adhesion, and possible ingrowth of blood vessels and/or bone tissue. It is worth mentioning that this uncontrolled pore orientation may lead to batch-to-batch variability influencing the consistency of results. In our experiments, a 15–60 μm pore diameter (Figure 1b) was chosen due to our previous successful experience with mesenchymal stem cells cultured in the pores of the same dimensions [29]. Such a diameter was also considered appropriate for the in vitro leukemia model since the size of cells ranged from 9 to 20 μm (Figure 3).

The superporous PHEMA hydrogel defined composition that was easy to control and replicable; the hydrogel was chemically and thermally stable, therefore sterilizable by ethanol or at high temperature, which made it suitable for cell culture. Moreover, nondegradability of PHEMA facilitated long-term incubations of cells in vitro. As the hydrogel porous structure did not collapse, it allowed the same support throughout the whole experiment. The hydrogel was easy to handle and could be transferred from well to well without any disruption.

All of the aforementioned aspects allowed long-term culture of HS-5, M2-10B4, MEC-1, and HG-3 cell lines in the P(HEMA-AEMA)-RGDS hydrogels for a minimum of 70 days (Figure 5a–d). The observed increase of metabolic activity of the cell line culture by day 20 (Figure 5a,b,d) could be explained by their log or lag phase of growth. On day 20, the cell lines probably reached their plateau, i.e., stationary phase, as well as excessive confluence, which temporarily led to the inhibition of contacts and increased death rate, hence, lower metabolic activity. Since the manipulation with 3D-cultured cell lines only involved medium exchange and no dilution, the cell lines had to develop their own mechanisms of coping with higher cell densities that exceeded medium capacity, e.g., by forming clumps that detached from the pores of scaffold and created space for the growth of new cells. Other adverse effects affecting the metabolic activity of cells might be following: (i) repeated exposure to AlamarBlue™ and its retention in the hydrogels, which could negatively influence cell viability, and metabolic activity; (ii) medium exchange that occurred every 2 to 3 days; thus, it depended on whether the measurement was performed after 2- or 3-day incubation. However, as the cancer cell line resilience dominated over the adverse effects, their metabolic activity mostly returned to the previous rate, which could be seen after 40–50 days, when the growth rate probably slightly exceeded the death rate.

Contrary to other cell lines, B-CLLs only have a short lifespan (Figure 5e; Figure 6 and Figure 7). However, the RGDS peptide proved to be essential for the cell-substrate adhesion of B-CLLs, since the initial number of seeded cells, their metabolic activity, and viability were higher in RGDS-modified scaffolds compared to unmodified ones (Figure 4a–c; Figure 5e). Contrary to our expectations, the 3D culture itself did not affect prolongation of B-CLL survival, as their metabolic activity as well as viability dropped within 4–7 days (Figure 5e; Figure 6 and Figure 7), which can be explained by numerous theories suggested below.

The size of the pores, as well as their orientations and the process of cell seeding, led to the formation of cell clumps inside of the hydrogels (Figure 4b,c). The presence of the cell clumps can be beneficial, since the cells are in close contact, allowing efficient signal exchange and short-distance transport of growth factors. On the other hand, such proximity may later result in a deteriorated export of the cellular wastes from the pores, accounting for nutrient and oxygen deficiency. Although such hypoxia and malnutrition conditions are usually observed in the center of solid tumors or the cancer-cell spheroids in vitro, they induce abundant cell death, such as central necrosis [12], which may be one of the reasons why primary B-CLL metabolic activity dropped so fast. To avoid the diffusion-limited tumor growth, the B-CLLs could be dynamically cultured under direct perfusion flow [35], in capillary-like hollow fibers [36], or bioreactors using microgravity [10]. These might enhance the transport of medium components and also mechanically disrupt large clumps; nevertheless, such methods require special equipment.

Another explanation for reduced cell viability and metabolic activity is methodological. It should be noted that AlamarBlue™ is nontoxic for short periods, but cytotoxic in long time intervals (24–72 h) [37]. Thus, repeated exposure combined with resazurin retention in the hydrogels may have eventually damage the primary B-CLLs. The same applies to Sytox green, which was earlier considered as a nonpermeable and nontoxic fluorescent dye; however, its toxic effect was only evaluated during a 3-day incubation [38].

Surprisingly, coculture with M2-10B4, soluble IL4, or CD40L did not affect B-CLL survival, which is in disagreement with the existing literature [34,39]. The results suggest the distribution of the growth factors (either artificially supplemented or naturally produced by the cocultured cells) was insufficient under the tested culture conditions, or that the CLLs did not respond to the external stimuli provided by the BMSCs and the supplemented additives. To increase the probability of molecule delivery, peptides such as CD40L and IL-4 could be promising for direct immobilization on the scaffolds, since it has been already proven that, e.g., immobilized anti-immunoglobulin M provided a more potent CLL stimulus than a soluble one [40]. A boosted delivery of growth factors could also be achieved by transfecting BMSCs with vectors that encode human CD40L and IL-4, considering that B-CLLs and BMSCs tend to grow in proximity. Such a transfer might be preferable, assuming that membrane-bound CD40L shows a superior capacity to activate CD40 signaling and resembles natural interactions, where CD40L is probably delivered by cell–cell contacts [41].

Furthermore, patient variability (Table S1) did not cause differences between B-CLL in vitro survival (Figure 5e; Figure 6 and Figure 7), indicating that the CLL severity might have no impact on the B-CLL viability ex vivo, and/or there is a prevailing negative effect inducing B-CLL apoptosis before the differences can manifest. Let us emphasize that due to required high amount of cells used for the seeding (~50 million/scaffold), the results shown in Figure 5, Figure 6 and Figure 7 only include a low number of replicates (n = 3). More research would be needed to identify and clarify any adverse effects.

Despite all the limitations, the RGDS-immobilized P(HEMA-AEMA) hydrogels can serve as a platform for studying B-CLL biology in vitro due to their partial transparency, which facilitates time-lapse studies of B-CLL migration or monitoring their interactions with adjacent cells in 3D. In addition, once the observed growth-specific vulnerabilities are clarified at the molecular level, they can potentially serve as therapeutic targets [42]. Last but not least, it was demonstrated that the direct DNA isolation from scaffold-cultured cells is efficient, which allows deep analysis, including sequencing. In the case of coculture with BMSCs, lines of nonhuman origin (e.g., M2-10B4) have to be used, to make the DNA from B-CLLs distinguishable by species-specific primers. Even though this adds one more step to the DNA analysis, the method is preferable to scaffold decellularization followed by cell sorting, since both these steps are also accompanied by high losses of cells, lowering thus DNA yield.

## 4. Materials and Methods

### 4.1. Materials

HEMA, EDMA, AEMA, AIBN, GMBS, ammonium oxalate, Ficoll^®^ Paque Plus, penicillin-streptomycin (P/S), and sterile trypsin with 0.1% disodium ethylenediaminetetraacetate were obtained from Sigma-Aldrich (St. Luis, MO, USA). 1,4-Dioxane was from Fluka (Buchs, Switzerland). Phosphate buffer saline (PBS) was prepared from Na_2_HPO_4_ and KH_2_PO_4_. In-house prepared ammonium oxalate (LachNer; Neratovice, CR) crystals were obtained by repeated crystallization from aqueous solution at 50 °C with cooling to 23 °C; the crystals were then classified in saturated oxalate solution on a sieve (0.4 × 15 mm mesh). Calcein acetoxymethyl ester (AM), propidium iodide, Hoechst 33342, AlamarBlue™ cell viability reagent, CellTrace™ violet cell proliferation kit, and Sytox™ green nucleic acid stain were purchased from Thermo Fisher Scientific (Eugene, OR, USA). Reagents for peptide synthesis were from Iris Biotech (Marktredwitz, Germany). Dulbecco’s Modified Eagle Medium (DMEM) with stable glutamine and sodium pyruvate, Iscove’s Modified Dulbecco’s Medium (IMDM) with stable glutamine and 25 mM 2-[4-(2-hydroxy-ethyl)piperazin-1-yl]ethanesulfonic acid (HEPES), RPMI 1640 medium with stable glutamine and 25 mM HEPES, and fetal bovine serum (FBS) were from Biosera (Nuaille, France). Recombinant human IL-4 and sCD40 ligand were purchased from Peprotech (Rocky Hill, NJ, USA). RosetteSep™ human B cell enrichment cocktail and CD3+ cell depletion cocktail were supplied by Stemcell™ Technologies (Vancouver, Canada). FastDNA sample spin kit for soil was obtained from MP Biomedicals (Santa Ana, CA, USA) and the genomic DNA screen tape was from Agilent (Santa Clara, CA, USA). An acridine orange/propidium iodide cell viability kit was supplied by Logos Biosystems (Anyang, Korea). Other chemicals were purchased from LachNer. Q-water was ultrafiltered on a Milli-Q Gradient A10 apparatus (Millipore; Molsheim, France).

Cell culture plates and flasks were manufactured by Techno Plastic Products (Trasadingen, Switzerland). The OptiPlate-96 White microplates were made by PerkinElmer (Waltham, MA, USA).

### 4.2. Synthesis of P(HEMA-AEMA) Scaffolds

HEMA (9.8 g), EDMA (0.1 g), AEMA (0.1 g), and AIBN (40 mg) were dissolved in 1,4-dioxane (5 mL) to obtain the monomer phase. Injection syringes (10 mL) were filled with ammonium oxalate crystals (4.33 mL) and the above-mentioned monomer phase (5.67 mL) and the mixture was polymerized at 60 °C for 16 h. Subsequently, the injection syringe was cut and the P(HEMA-AEMA) cylinder removed and washed with 10% aqueous ammonium chloride (60 mL) for three days to remove ammonium oxalate crystals. The cylinder was then transferred into water, washed with water ten times (50 mL each) for two days, and cut on cubes (4 × 4 mm), which were repeatedly washed with water until ammonium chloride was removed. The scaffolds were viewed with a Quanta FEG 200F scanning electron microscope (FEI; Brno, Czech Republic). The amount of nitrogen in the scaffolds was determined on a Perkin-Elmer 2400 CHN elemental analyzer (Norwalk, CT, USA).

### 4.3. Synthesis of Ac-CGGGRGDSGGGY-NH2 (RGDS) Peptide

The peptide was synthesized by a standard Fmoc/tBu solid-phase method on a TentaGel Rink Amide—R resin (0.18 mmol NH_2_/g; Rapp Polymere; Tuebingen, Germany). An automatic CEM Liberty Blue microwave peptide synthesizer (Matthews, NC, USA) and software version 1.31.5252.26519 were used with default *N,N*’-diisopropylcarbodiimide/Oxyma Pure coupling and piperidine deprotection cycles. To avoid aspartimide formation, the pseudoproline Asp-Ser [Fmoc-Asp(OtBu)-Ser(Psi(Me,Me)pro)-OH] building block was used for incorporation of aspartic and serine amino acids. The peptide was cleaved from the resin with a CF_3_COOH/triisopropylsilane/H_2_O mixture (95/2.5/2.5 *v*/*v*/*v*) and isolated by the precipitation in diethyl ether. The purification was performed on a preparative Knauer HPLC system (Berlin, Germany) equipped with a Knauer diode-array detector and Kinetex LC column (5 µm, C18, 1000 Å, 250 × 21.2 mm) using a gradient elution with a water/acetonitrile mixture containing 0.1% formic acid. The identity of the peptide was confirmed on a Bruker Ultrafle Extreme MALDI-TOF mass spectrometer (Bremen, Germany) and Advion expression L CMS mass detector (Ithaca, NY, USA).

### 4.4. Activation of P(HEMA-AEMA) Scaffolds and Immobilization of RGDS Peptide

In a 200 mL Erlenmeyer flask, P(HEMA-AEMA) cubes were washed with water/ethanol (7:3 *v*/*v*) mixture (15 mL), the liquid was sucked away, and the cubes were immersed in a solution (5 mL) prepared from 0.07 M phosphate buffer (pH 7.4; 7 mL) and 1,4-dioxane (3 mL). GMBS (5 mg) in 1,4-dioxane (0.5 mL) was added and the mixture was shaken (50 rpm) at room temperature (RT) for 30 min. The cubes were washed twice with ethanol (10 mL each), twice with water (10 mL each), and with 0.1 M phosphate buffer (pH 6.8; 10 mL). The buffer was withdrawn, and a solution of RGDS peptide (2 mg) in 0.1 M phosphate buffer (pH 6.8; 6 mL) was added. The reaction continued at RT for 75 min with shaking (50 rpm), and the resulting P(HEMA-AEMA)-RGDS cubes were washed with water five times (10 mL each), sterilized in 60% aqueous ethanol (20 mL) for 16 h, washed with water to remove ethanol, and stored at 4 °C.

### 4.5. Primary B-CLL Cells and Cell Lines

Human B-CLL cell lines, MEC-1 (DSMZ ACC 497; Braunschweig, Germany) [32], and HG-3 (DSMZ ACC 765) [43] were used, being the only commercially available in vitro proliferating B-CLLs. Vital primary B-CLLs were obtained from patients diagnosed with CLL and treated at the Department of Internal Medicine, Hematology and Oncology, University Hospital Brno, Czech Republic. The patients signed their informed consent in accordance with the Declaration of Helsinki under protocols approved by the Ethical Committee of the University Hospital Brno (date of approval: 4 April 2018). Primary B-CLLs were separated from the peripheral blood using Ficoll^®^ Paque Plus and Rosette Sep kits. The viability and number of cells directly after isolation (prior to cell culture) was evaluated in a Luna-FL™ dual fluorescence automated cell counter (Logos Biosystems; Anyang, South Korea) with an acridine orange/propidium iodide cell viability kit.

In order to mimic the microenvironment of the bone marrow, the B-CLL cells were cultured with adherent BMSCs, which support B-CLL survival in vitro [22,44]. BMSCs included human HS-5 cell line (CRL-11882™; ATCC; Manassas, VA, USA) [45] and murine M2-10B4 cell line (CRL-1972™; ATCC) [46].

For characterization of cell line sources, see Supporting Materials (SMs), Table S5. Patient’s samples are described in Table S1.

### 4.6. Cell Culture Conditions

Cell cultures were incubated at 37 °C in a 5% CO_2_ atmosphere in culture flasks in the medium recommended by the ATCC or DMSZ bioresource center and supplemented with 10% FBS and 1% P/S; medium was regularly exchanged two or three times a week. HS-5 or M210B4 were cultured in DMEM, MEC-1 in IMDM, and HG-3 or primary B-CLLs were incubated in RPMI 1640. Bone marrow stromal cells and primary B-CLLs were cocultured in RPMI 1640 medium supplemented with 20% FBS. In some cases, the cell suspensions were continuously shaken (30 rpm) on a Rocker 25 apparatus (Labnet International; Big Flats, NY, USA) at 37 °C under 5% CO_2_ atmosphere. When the cell metabolic activity or viability was studied in the presence of CD40L and/or IL-4, the cells were stimulated continuously, as a single stimulation was insufficient to maintain cell division, survival, and differentiation [39].

### 4.7. Seeding of Cells in P(HEMA-AEMA) Scaffolds

**Figure 9:**
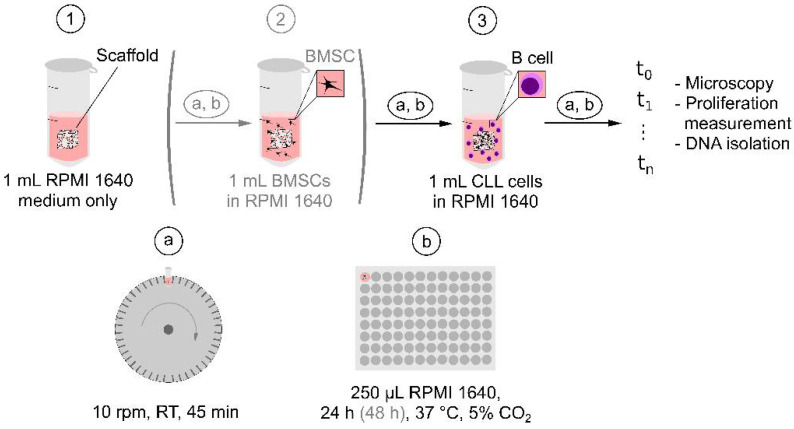
Schematic view of B cell (optionally BMSC—see the brackets) seeding in P(HEMA-AEMA) scaffold. Each imbibing (1) or seeding (2, 3) step was performed by placing the scaffold into the microtube with medium (1) or cell suspension (2, 3) followed by (**a**) rotation of the microtube around horizontal axis, and (**b**) transferring the scaffold into fresh medium in 96-well plate.

Cells were seeded in the P(HEMA-AEMA) hydrogels according to a technique modified from an earlier published report [47]; schematic view of the seeding is shown in Figure 9. Prior to the seeding, the hydrogel cubes were placed in 2 mL round-bottom tubes filled with the corresponding medium (1 mL) and rotated (10 rpm; LabRoller II H5100; Labnet International; Big Flats, NY, USA) around the horizontal axis at RT for 45 min. Each scaffold was placed in the medium (250 μL) in the 96-well plate and cultured at 37 °C for 24 h under 5% CO_2_ atmosphere. For BMSCs and B-CLLs coculture, the medium-imbibed cubes were placed in the BMSC suspension (1 mL) under the above-described rotation and culturing continued for 48 h. Finally, the freshly isolated B-CLL cell suspension (1 mL) with initial cell viability >98% was seeded in the scaffold with rotation and cultured in the medium in a 96-well plate for several days under continuous monitoring. The scaffolds were regularly (within one to three days) transferred to new wells containing fresh medium (250 μL each), always without any passaging, i.e., trypsinization.

### 4.8. Cell Imaging and Image Analysis

For single time point measurements, including analysis of cell distribution and morphology, live and dead cells and nuclei of the 3D-cultured cells were stained with calcein AM (1:1000), propidium iodide (1:1000), and Hoechst 33342 (1:2000), respectively; these dyes are toxic, thus terminating the experiment [38,48,49]. To continuously monitor the 3D cell culture, the cells were initially stained with CellTrace violet and then with Sytox green (1:1000) each day, which was followed by washing with PBS (pH 7.4) three times. In both cases, the dyes were diluted in the corresponding medium with 1% P/S (without FBS) and the P(HEMA-AEMA)-RGDS cubes with incubated cells were kept at 37 °C for 45 min in the dark. The scaffolds were transferred in PBS (50 μL) and observed using a Zeiss LSM-800 confocal microscope (Jena, Germany) with z-axis stacking to scan multiple scaffold layers and tile scanning of adjacent positions in the x–y axis. At each time point, 10 square layers (area 8.64 mm^2^) were scanned to the depth of 135 μm. After the acquisition, the images were postprocessed using ZEN and Fiji software (see Appendix A) [50].

### 4.9. Metabolic Activity

Metabolic activity was determined using AlamarBlue™ cell viability reagent diluted in 1:10 ratio (*v*/*v*) in complete medium with 10% FBS and 1% P/S. In a 96-well culture plate, diluted AlamarBlue™ reagent (150 μL) was added per well, each containing one P(HEMA-AEMA) cube. Incubation continued at 37 °C for 3 h in the dark, reagent (100 μL) surrounding the hydrogel was transferred into an OptiPlateTM 96-well plate without the lid, and fluorescence was measured using a Spark 10 M multimode microplate reader (Tecan; Männedorf, Switzerland) at 530 and 590 nm (excitation and emission, respectively). As AlamarBlue™ can interact with the cell culture medium, resulting in inconsistent fluorescence signal [51], P(HEMA-AEMA) cubes without the cells, but treated according to the same protocol, were used as a negative control. After the incubation with the reagent, the scaffolds were washed in PBS (pH 7.4) and put into a corresponding fresh complete medium.

### 4.10. DNA Isolation and Quality Control

DNA was isolated by FastDNA sample SPIN kit for soil according to the manufacturer’s protocol 24 h after seeding the scaffolds with B-CLLs [52]. The following modifications were introduced to the protocol: homogenization was carried out for 8 min at 2000 rpm (Thermomixer C; Eppendorf; Hamburg, Germany); in the elution step, incubation continued at 55 °C for 5 min. The DNA quality was controlled by electrophoresis using a 4200 TapeStation system (Agilent; Santa Clara, CA, USA) with the genomic DNA screen tape as directed in the manual [53].

### 4.11. Statistical Analysis

Graphs were plotted using GraphPad Prism software, version 8.4.3 for Windows (San Diego, CA, USA). Vertical bars in the graphs denoted a 95% confidence interval. Statistical analysis was performed using Statistica, version 13.5.0.17 for Windows (Statsoft; Tulsa, OK, USA) with two-way ANOVA corrected for multiple comparisons, or using computing environment R [54] and ImerTest package, type III analysis of variance with Satterthwaite’s method. *p* values < 0.05 were considered significant.

## 5. Conclusions

In the artificial scaffolds, pore size and type of pore structure play a key role in cell behavior. Interconnecting pores promote the removal of wastes, transport of nutrients, and facilitate proliferation and migration of the cells. This report demonstrates that the P(HEMA-AEMA)-RGDS hydrogel supported long-term viability and metabolic activity of B-CLL cell lines (MEC-1 and HG-3) and bone marrow stromal cell lines (HS-5 and M2-10B4), which mimic the microenvironment of bone marrow. This was confirmed by both confocal microscopy and AlamarBlue™ assay. Moreover, the culture of the primary B-CLLs was performed, confirming that they survived in the 3D in vitro model for 4–6 days, independently on the cocultured cells or growth factors in the media. RGDS modification turned out to be beneficial for the survival of the B-CLLs in the culture, since it increased the probability of the cells to be seeded into the scaffold pores, promoting the cell–cell and cell–surface interactions. This is an important prerequisite for creating a 3D model of CLL, which could serve as a leukemia drug-testing platform that is much more realistic than the presently used 2D systems. As such, this model cannot generally replace phase II clinical trials, but it can supplement the use of animal in vivo protocols since it can exclude poor drug candidates, e.g., toxic and malfunctional ones, and thus lower financial and time costs. Our protocol for seeding the cells into the scaffolds does not apply for in vitro tests only, but it can be hypothetically extended to the xenograft models after transferring the scaffolds into immunodeficient animals (mice, rats, etc.). Additionally, the system can be subjected to thorough DNA analysis, as we introduced a novel methodology for DNA isolation of the 3D cocultured cells. Our new 3D model based on a RGDS-modified PHEMA hydrogel thus contributes to solving the etiology of chronic lymphocytic leukemia, a disease which remains incurable.

## Data Availability

All data presented in this study are contained within this article or its supplementary materials. Further details are available upon request from the corresponding author.

## Supplementary Materials

The following are available online at https://www.mdpi.com/1422-0067/22/5/2376/s1: Table S1: Characterization of patients’ samples; Table S2: Statistical evaluation of the effect of modification with RGDS peptide and plate shaking on cellular metabolic activity; Table S3: Statistical evaluation of coculture with M210B4 supported with medium flow on primary CLL cells (B-CLLs) survival; Table S4: Statistical evaluation of the effect of the interleukin 4 (IL-4) and CD40 ligand (CD-40L) added into shaken and unshaken medium on primary B-CLL metabolic activity; Table S5: Characterization of cell line sources.

## Appendix A

### Postprocessing of Microscopy Images

The images were postprocessed using ZEN build-in plug-ins. Multiple tiles in the x–y axis were combined with a stitching tool and the 3D image was converted into the 2D one by orthogonal projection, selecting the pixels with the maximum intensity from all the layers in the z-axis. The images were then processed using the open source Fiji software. Prior to counting, the image was converted to the mask using following plug-ins: (i) the background noise was removed and signal of overexposed areas was lowered by the “subtract background” plug-in, (ii) the image was converted to 8-bit by the “adjust threshold” plug-in, choosing the optimal threshold manually to avoid omission of any cell, (iii) the “watershed” plug-in drew a 1-pixel line between adjacent cells growing in the clusters to count each cell and not the clump, and (iv) the “analyze particle” plug-in counted the particles above the defined size (10-infinity).

## References

[1] Kipps T.J., Stevenson F.K., Wu C.J., Croce C.M., Packham G., Wierda W.G., O’Brien S., Gribben J., Rai K. (2017). Chronic Lymphocytic Leukaemia. Nat. Rev. Dis. Primer.

[2] Hallek M. (2019). Chronic Lymphocytic Leukemia: 2020 Update on Diagnosis, Risk Stratification and Treatment. Am. J. Hematol..

[3] Gribben J.G. (2010). How I Treat CLL up Front. Blood.

[4] Bosch F., Dalla-Favera R. (2019). Chronic Lymphocytic Leukaemia: From Genetics to Treatment. Nat. Rev. Clin. Oncol..

[5] Satpathy A., Datta P., Wu Y., Ayan B., Bayram E., Ozbolat I.T. (2018). Developments with 3D Bioprinting for Novel Drug Discovery. Expert Opin. Drug Discov..

[6] Chew S.A., Moscato S., George S., Azimi B., Danti S. (2019). Liver Cancer: Current and Future Trends Using Biomaterials. Cancers.

[7] Li D., Lin T.L., Lipe B., Hopkins R.A., Shinogle H., Aljitawi O.S. (2018). A Novel Extracellular Matrix-Based Leukemia Model Supports Leukemia Cells with Stem Cell-like Characteristics. Leuk. Res..

[8] Zhang C., Yang Z., Dong D.-L., Jang T.-S., Knowles J.C., Kim H.-W., Jin G.-Z., Xuan Y. (2020). 3D Culture Technologies of Cancer Stem Cells: Promising Ex Vivo Tumor Models. J. Tissue Eng..

[9] Burger J.A., Gribben J.G. (2014). The Microenvironment in Chronic Lymphocytic Leukemia (CLL) and Other B Cell Malignancies: Insight into Disease Biology and New Targeted Therapies. Semin. Cancer Biol..

[10] Barbaglio F., Belloni D., Scarfò L., Sbrana F.V., Ponzoni M., Bongiovanni L., Pavesi L., Zambroni D., Stamatopoulos K., Caiolfa V.R. (2020). 3D Co-Culture Model of Chronic Lymphocytic Leukemia Bone Marrow Microenvironment Predicts Patient-Specific Response to Mobilizing Agents. Haematologica.

[11] Dos Santos J., Enfield L., Dos Santos S.B., Allenby M.C., Zemenides S., Mantalaris A., Panoskaltsis N. (2017). Primary Chronic Lymphocytic Leukemia Cells Can Be Maintained Long-Term in Serum-Free, Cytokine-Free 3D Culture. Blood.

[12] Verjans E.-T., Doijen J., Luyten W., Landuyt B., Schoofs L. (2018). Three-Dimensional Cell Culture Models for Anticancer Drug Screening: Worth the Effort?. J. Cell. Physiol..

[13] Datta P., Dey M., Ataie Z., Unutmaz D., Ozbolat I.T. (2020). 3D Bioprinting for Reconstituting the Cancer Microenvironment. Npj Precis. Oncol..

[14] El-Sherbiny I.M., Yacoub M.H. (2013). Hydrogel Scaffolds for Tissue Engineering: Progress and Challenges. Glob. Cardiol. Sci. Pract..

[15] Dhandayuthapani B., Yoshida Y., Maekawa T., Kumar D.S. (2011). Polymeric Scaffolds in Tissue Engineering Application: A Review. Int. J. Polym. Sci..

[16] Lee J., Cuddihy M.J., Kotov N.A. (2008). Three-Dimensional Cell Culture Matrices: State of the Art. Tissue Eng. Part B Rev..

[17] Drury J.L., Mooney D.J. (2003). Hydrogels for Tissue Engineering: Scaffold Design Variables and Applications. Biomaterials.

[18] Zhu J., Marchant R.E. (2011). Design Properties of Hydrogel Tissue-Engineering Scaffolds. Expert Rev. Med. Devices.

[19] Atzet S., Curtin S., Trinh P., Bryant S., Ratner B. (2008). Degradable Poly(2-Hydroxyethyl Methacrylate)-*co*-Polycaprolactone Hydrogels for Tissue Engineering Scaffolds. Biomacromolecules.

[20] Kůdela J. (1987). Hydrogels. Encyclopedia of Polymer Science and Technology.

[21] ten Hacken E., Burger J.A. (2014). Microenvironment Dependency in Chronic Lymphocytic Leukemia: The Basis for New Targeted Therapies. Pharmacol. Ther..

[22] Lagneaux L., Delforge A., Bron D., De Bruyn C., Stryckmans P. (1998). Chronic Lymphocytic Leukemic B Cells but Not Normal B Cells Are Rescued from Apoptosis by Contact with Normal Bone Marrow Stromal Cells. Blood.

[23] Crassini K., Shen Y., Mulligan S., Giles Best O. (2017). Modeling the Chronic Lymphocytic Leukemia Microenvironment in Vitro. Leuk. Lymphoma.

[24] Jabs J., Zickgraf F.M., Park J., Wagner S., Jiang X., Jechow K., Kleinheinz K., Toprak U.H., Schneider M.A., Meister M. (2017). Screening Drug Effects in Patient-Derived Cancer Cells Links Organoid Responses to Genome Alterations. Mol. Syst. Biol..

[25] Sommerová L., Michalová E., Hrstka R. (2018). New approaches for chemosensitivity testing in malignant diseases. Klin. Onkol. Cas. Ceske Slov. Onkol. Spolecnosti.

[26] Lee J., Li M., Milwid J., Dunham J., Vinegoni C., Gorbatov R., Iwamoto Y., Wang F., Shen K., Hatfield K. (2012). Implantable Microenvironments to Attract Hematopoietic Stem/Cancer Cells. Proc. Natl. Acad. Sci. USA.

[27] Turnbull G., Clarke J., Picard F., Riches P., Jia L., Han F., Li B., Shu W. (2018). 3D Bioactive Composite Scaffolds for Bone Tissue Engineering. Bioact. Mater..

[28] Kubinová Š., Horák D., Syková E. (2009). Cholesterol-Modified Superporous Poly(2-Hydroxyethyl Methacrylate) Scaffolds for Tissue Engineering. Biomaterials.

[29] Macková H., Plichta Z., Proks V., Kotelnikov I., Kučka J., Hlídková H., Horák D., Kubinová Š., Jiráková K. (2016). RGDS- and SIKVAVS-Modified Superporous Poly(2-Hydroxyethyl Methacrylate) Scaffolds for Tissue Engineering Applications. Macromol. Biosci..

[30] Singh S., Ghode S., Devi M.R., Limaye L., Kale V. (2015). Phenotypic and Functional Characterization of a Marrow-Derived Stromal Cell Line, M210B4 and Its Comparison with Primary Marrow Stromal Cells. Biomed. Res. J..

[31] Kurtova A.V., Balakrishnan K., Chen R., Ding W., Schnabl S., Quiroga M.P., Sivina M., Wierda W.G., Estrov Z., Keating M.J. (2009). Diverse Marrow Stromal Cells Protect CLL Cells from Spontaneous and Drug-Induced Apoptosis: Development of a Reliable and Reproducible System to Assess Stromal Cell Adhesion-Mediated Drug Resistance. Blood.

[32] Stacchini A., Aragno M., Vallario A., Alfarano A., Circosta P., Gottardi D., Faldella A., Rege-Cambrin G., Thunberg U., Nilsson K. (1999). MEC1 and MEC2: Two New Cell Lines Derived from B-Chronic Lymphocytic Leukaemia in Prolymphocytoid Transformation. Leuk. Res..

[33] German Collection of Microorganisms and Cell Cultures GmbH: Details. https://www.dsmz.de/collection/catalogue/details/culture/ACC-765.

[34] Ghia P., Circosta P., Scielzo C., Vallario A., Camporeale A., Granziero L., Caligaris-Cappio F. (2005). Differential effects on CLL cell survival exerted by different microenvironmental elements. Chronic Lymphocytic Leukemia.

[35] Bourgine P.E., Klein T., Paczulla A.M., Shimizu T., Kunz L., Kokkaliaris K.D., Coutu D.L., Lengerke C., Skoda R., Schroeder T. (2018). In Vitro Biomimetic Engineering of a Human Hematopoietic Niche with Functional Properties. Proc. Natl. Acad. Sci. USA.

[36] Walsby E., Buggins A., Devereux S., Jones C., Pratt G., Brennan P., Fegan C., Pepper C. (2014). Development and Characterization of a Physiologically Relevant Model of Lymphocyte Migration in Chronic Lymphocytic Leukemia. Blood.

[37] Nakayama G.R., Caton M.C., Nova M.P., Parandoosh Z. (1997). Assessment of the Alamar Blue Assay for Cellular Growth and Viability in Vitro. J. Immunol. Methods.

[38] Chiaraviglio L., Kirby J.E. (2014). Evaluation of Impermeant, DNA-Binding Dye Fluorescence as a Real-Time Readout of Eukaryotic Cell Toxicity in a High Throughput Screening Format. Assay Drug Dev. Technol..

[39] Rush J.S., Hodgkin P.D. (2001). B Cells Activated via CD40 and IL-4 Undergo a Division Burst but Require Continued Stimulation to Maintain Division, Survival and Differentiation. Eur. J. Immunol..

[40] Rombout A., Lust S., Offner F., Naessens E., Verhasselt B., Philippé J. (2016). Mimicking the Tumour Microenvironment of Chronic Lymphocytic Leukaemia in Vitro Critically Depends on the Type of B-Cell Receptor Stimulation. Br. J. Cancer.

[41] Natoni A., O’Dwyer M., Santocanale C. (2013). A Cell Culture System That Mimics Chronic Lymphocytic Leukemia Cells Microenvironment for Drug Screening and Characterization. Methods Mol. Biol. Clifton NJ.

[42] Han K., Pierce S.E., Li A., Spees K., Anderson G.R., Seoane J.A., Lo Y.-H., Dubreuil M., Olivas M., Kamber R.A. (2020). CRISPR Screens in Cancer Spheroids Identify 3D Growth-Specific Vulnerabilities. Nature.

[43] Rosén A., Bergh A.-C., Gogok P., Evaldsson C., Myhrinder A.L., Hellqvist E., Rasul A., Björkholm M., Jansson M., Mansouri L. (2012). Lymphoblastoid Cell Line with B1 Cell Characteristics Established from a Chronic Lymphocytic Leukemia Clone by in Vitro EBV Infection. Oncoimmunology.

[44] Crompot E., Van Damme M., Pieters K., Vermeersch M., Perez-Morga D., Mineur P., Maerevoet M., Meuleman N., Bron D., Lagneaux L. (2017). Extracellular Vesicles of Bone Marrow Stromal Cells Rescue Chronic Lymphocytic Leukemia B Cells from Apoptosis, Enhance Their Migration and Induce Gene Expression Modifications. Haematologica.

[45] Roecklein B.A., Torok-Storb B. (1995). Functionally Distinct Human Marrow Stromal Cell Lines Immortalized by Transduction with the Human Papilloma Virus E6/E7 Genes. Blood.

[46] Lemoine F.M., Humphries R.K., Abraham S.D., Krystal G., Eaves C.J. (1988). Partial Characterization of a Novel Stromal Cell-Derived Pre-B-Cell Growth Factor Active on Normal and Immortalized Pre-B Cells. Exp. Hematol..

[47] Thevenot P., Nair A., Dey J., Yang J., Tang L. (2008). Method to Analyze Three-Dimensional Cell Distribution and Infiltration in Degradable Scaffolds. Tissue Eng. Part C Methods.

[48] Jonsson B., Liminga G., Csoka K., Fridborg H., Dhar S., Nygren P., Larsson R. (1996). Cytotoxic Activity of Calcein Acetoxymethyl Ester (Calcein/AM) on Primary Cultures of Human Haematological and Solid Tumours. Eur. J. Cancer Oxf. Engl. 1990.

[49] Durand R.E., Olive P.L. (1982). Cytotoxicity, Mutagenicity and DNA Damage by Hoechst 33342. J. Histochem. Cytochem. Off. J. Histochem. Soc..

[50] Schindelin J., Arganda-Carreras I., Frise E., Kaynig V., Longair M., Pietzsch T., Preibisch S., Rueden C., Saalfeld S., Schmid B. (2012). Fiji: An Open-Source Platform for Biological-Image Analysis. Nat. Methods.

[51] Munshi S., Twining R.C., Dahl R. (2014). Alamar Blue Reagent Interacts with Cell-Culture Media Giving Different Fluorescence over Time: Potential for False Positives. J. Pharmacol. Toxicol. Methods.

[52] FastDNATM SPIN Kit for Soil, MP Biomedicals—Instruction Manual. https://media.mpbio.com/productattachment/LS082019-EN-FastDNA-SPIN-Kit-for-Soil-116560200-Manual.pdf.

[53] Agilent Genomic DNA Screentape—Quick Guide for TapeStation Systems. https://www.agilent.com/cs/library/usermanuals/public/gDNA_QuickGuide.pdf.

[54] R Core Team (2020). R: A Language and Environment for Statistical Computing.

