# Supplementary material for: RGDS-Modified Superporous Poly(2-Hydroxyethyl Methacrylate)-Based Scaffolds as 3D In Vitro Leukemia Model
Source: Int J Mol Sci. 2021 Feb 27;22(5):2376. doi: 10.3390/ijms22052376 (PMC7956824; doi:10.3390/ijms22052376)
Supplement: Supplementary file 1 [file ijms-22-02376-s001.pdf]

Supplementary Materials

Table S1. Characterization of patient's samples.

Patient No.	Age	Sex	Leu ($\times 10^9$/l)	Lym (%)	*IGHV* mutational status	*TP53* mutational status	Cytogenetic aberrations and other prognostically relevant molecular changes	Years after CLL diagnosis	Treatment
1	83	Female	93.7	92.8	Unmutated	Wild type	-X;<br>+12	9	1. BR<br>2. Rituximab, chlorambucil
2	75	Male	162.0	87.4	Unmutated	Wild type	ZAP70 positive;<br>RB1 deletion	10	1. Reduced BR
3	62	Male	61.6	90.5	Unmutated	Mutated cumulatively in 57.25 % cells	del13q;<br>del11q	7	1. Reduced FCR<br>2. Lumbar spine radiotherapy<br>3. BR<br>4. Acalabrutinib
4	66	Female	123.0	93.4	Unmutated	Deletion in 22 % cells;<br>mutated cumulatively in 34.83 % cells	8q24/MYC rearrangement	4	1. Alemtuzumab<br>2. Ibrutinib
5	58	Male	77.4	86.7	Unmutated	Wild type	del13q14	2	None
6	65	Female	59.0	85.8	Mutated	Wild type	Biclonal CLL	8	None
7	83	Female	108.0	92.2	Unmutated	Mutated cumulatively in 91 % cells	13q14 biallelic deletion	9	1. Alemtuzumab<br>2. Prednisonum

Leu – leukocytes; Lym – lymphocytes; *IGHV* – immunoglobulin heavy variable gene; *TP53* – tumor protein 53 gene; CLL – chronic lymphocytic leukemia; ZAP70 – zeta chain of T cell receptor associated protein kinase 70; RB1 – retinoblastoma protein 1; MYC – mylocytomatosis oncogene; BR – bendamustine, rituximab; FCR – fludarabine, cyclophosphamide, rituximab.

International Journal of Molecular Sciences

MDPI

Table S2. Statistical evaluation of the effect of modification with RGDS peptide and plate shaking on cellular metabolic activity.

Cells	Probability (*p*)	
	RGDS	Shaking
HS-5	0.451270	0.000014
M2-10B4	0.322289	0.009031
MEC-1	0.248244	0.083556
HG-3	0.743284	0.197314
Primary B-CLLs	0.000003	0.890298

Significant values are in red. B-CLLs – chronic lymphocytic leukemia cells.

Table S3. Statistical evaluation of co-culture with M210B4 supported with medium flow on primary CLL cells (B-CLLs) survival.

Effect	Probability (*p*)
M2-10B4 co-culture	0.13695
Shaking	0.50816

Table S4. Statistical evaluation of the effect of the interleukin 4 (IL-4) and CD40 ligand (CD-40L) added into shaken and unshaken medium on primary B-CLLs metabolic activity.

Effect	Probability (*p*)
CD40L	0.8099767
IL-4	0.3813562
Shaking	0.0522501

Table S5. Characterization of cell line sources.

Cell line	Source	Reference
MEC-1	Established from the peripheral blood of a 61-year-old Caucasian man with chronic lymphocytic leukemia (CLL) transformed to B-cell prolymphocytic leukemia (B-PLL)	[1]
HG-3	Established from leukemic B cells of a 70-year-old Caucasian man with CLL at the time of diagnosis staged as Rai II	[2]
HS-5	Established from a 30-year-old Caucasian healthy man	[3]
M2-10B4	Derived from bone marrow stromal cells from a (C57BL/6J X C3H/HeJ)F1 mouse	[4]

References

1. Stacchini, A.; Aragno, M.; Vallario, A.; Alfarano, A.; Circosta, P.; Gottardi, D.; Faldella, A.; Rege-Cambrin, G.; Thunberg, U.; Nilsson, K.; et al. MEC1 and MEC2: Two New Cell Lines Derived from B-Chronic Lymphocytic Leukaemia in Prolymphocytoid Transformation. *Leuk. Res.* **1999**, *23*, 127–136, doi:10.1016/s0145-2126(98)00154-4.
2. Rosén, A.; Bergh, A.-C.; Gogok, P.; Evaldsson, C.; Myhrinder, A.L.; Hellqvist, E.; Rasul, A.; Björkholm, M.; Jansson, M.; Mansouri, L.; et al. Lymphoblastoid Cell Line with B1 Cell Characteristics Established from a Chronic Lymphocytic Leukemia Clone by in Vitro EBV Infection. *Oncoimmunology* **2012**, *1*, 18–27, doi:10.4161/onci.1.1.18400.
3. Roecklein, B.A.; Torok-Storb, B. Functionally Distinct Human Marrow Stromal Cell Lines Immortalized by Transduction with the Human Papilloma Virus E6/E7 Genes. *Blood* **1995**, *85*, 997–1005.
4. Lemoine, F.M.; Humphries, R.K.; Abraham, S.D.; Krystal, G.; Eaves, C.J. Partial Characterization of a Novel Stromal Cell-Derived Pre-B-Cell Growth Factor Active on Normal and Immortalized Pre-B Cells. *Exp. Hematol.* **1988**, *16*, 718–726.
